# Hippocampal abnormality and response to vagus nerve stimulation in epilepsy

**DOI:** 10.1111/epi.18658

**Published:** 2025-10-03

**Authors:** Harry J. Clifford, Sonja Fenske, Jonathan Horsley, Callum Simpson, Nathan Evans, Yujiang Wang, Tiago da Silva Costa, Rhys H. Thomas, Sabahat Iqbal, Cameron A. Elliott, John S. Duncan, Peter N. Taylor

**Affiliations:** ^1^ CNNP Lab, Interdisciplinary Computing and Complex BioSystems Group, School of Computing Newcastle University Newcastle upon Tyne UK; ^2^ Faculty of Medical Sciences Newcastle University Newcastle upon Tyne UK; ^3^ UCL Queen Square Institute of Neurology London UK; ^4^ Northern Centre for Mood Disorders Newcastle University Newcastle upon Tyne UK; ^5^ Cumbria, Northumberland, Tyne and Wear NHS Foundation Trust Newcastle upon Tyne UK; ^6^ National Institute for Health and Care Research (NIHR) Newcastle Biomedical Research Centre Newcastle upon Tyne UK; ^7^ Neurosciences Royal Victoria Infirmary Newcastle‐upon‐Tyne UK; ^8^ Department of Brain Sciences Imperial College London London UK; ^9^ Division of Neurosurgery, Department of Surgery University of Alberta Edmonton Alberta Canada

**Keywords:** biomarker, MRI, prediction

## Abstract

Vagus nerve stimulation (VNS) reduces seizure frequency and severity in some, but not all, individuals with epilepsy. The hippocampus has been implicated in VNS response, but is yet to be studied structurally using T1‐weighted (T1w) magnetic resonance imaging (MRI). In this study we hypothesized greater hippocampal abnormality in VNS non‐responders. Using hippocampal morphometrics, we extracted the volumes of four hippocampal regions from T1w MRI across three groups; VNS responders (n=42), non‐responders (n=50), and healthy controls (n=100). We first calculated the multivariate Mahalanobis distance using z‐scores from all four hippocampal regions to measure abnormality relative to controls. We then compared traditional univariate measures to the Mahalanobis distance. Response to VNS was defined as having a ≥50% seizure reduction 2 years post‐implantation. Hippocampal morphometrics were significantly more abnormal in non‐responders than responders (p=.005,biserialr=.32) using the multivariate Mahalanobis distance. Univariate approaches did not differ significantly between responders and non‐responders (p>.05). At the group level, non‐responders to VNS had greater structural hippocampal abnormality when using the multivariate approach. Conversely, this effect was lost with the univariate analysis. This suggests that abnormality is likely present in different parts of the hippocampus in different individuals. Future studies should incorporate multivariate, and potentially multi‐modal, information to better characterize the mechanisms of VNS response.

## 1 INTRODUCTION

Vagus nerve stimulation (VNS) is a common treatment for drug‐resistant epilepsy (DRE). VNS is a palliative therapy that attempts to reduce seizures but is unlikely to achieve seizure freedom. Therefore, VNS is used when surgical resection is infeasible or undesired to reduce seizure burden. Response to VNS is defined as a ≥50% reduction in seizure frequency after implantation, which is seen in around half of individuals.^1^, ^2^, ^3^ Given this response variability, and the lack of tools to guide the selection of individuals for implantation, a marker with predictive power would be valuable both clinically and mechanistically.^4^, ^5^

Studies have suggested that lesion identification on structural magnetic resonance imaging (MRI) correlates to response variability, but this association is unconfirmed.^6^, ^7^, ^8^, ^9^, ^10^ For example, Arya et al.^7^ reported that 62% of non‐responders had a lesion compared to 30% of responders. In contrast, Hava Ozlem et al.^8^ reported that 57% of non‐responders had a lesion compared to 70% of responders. A study of brain network characteristics derived from T1‐weighted (T1w) MRI showed a negative correlation between quantitative MRI brain abnormalities and reduction of seizure frequency.^11^ This variability between studies may reflect heterogeneity in lesion location and assessment methods.

A candidate location for VNS response prediction is the hippocampus. The hippocampus is connected directly to the thalamus, amygdala, and brainstem nuclei, which are affected directly by afferent fibers from the vagus nerve, and are all key components of the vagus afferent network (VagAN^12^). Previous studies found differences between response groups in hippocampal connectivity, both in functional (connectivity between the left hippocampus and both the right hippocampus and left thalamus)^13^ and diffusion‐weighted imaging (DWI; greater functional anisotropy in limbic system for responders).^14^ In addition, hippocampal abnormality is relatively common in DRE, and as such, is a component of the epileptogenic network for many individuals with epilepsy.

A univariate approach to evaluate brain abnormalities is powerful when a homogeneous effect is expected. Because the hippocampus contains several anatomic and functional sub‐regions, a multivariate approach could be beneficial if different sub‐regions are altered in different individuals. Recent studies in epilepsy have used the Mahalanobis distance for this purpose, demonstrating superiority to mass univariate testing when relating to epilepsy duration.^15^, ^16^

In this study we investigated whether hippocampal abnormality prior to VNS implantation is associated with VNS response at 2 years post‐implantation, using both multivariate and traditional univariate analyses. We hypothesized greater hippocampal abnormality in non‐responders to VNS.^4^

## 2 METHODS

### 2.1 Cohorts

We retrospectively identified individuals with epilepsy from the National Hospital for Neurology and Neurosurgery who had an implanted vagus nerve stimulator found in hospital records. We excluded individuals without T1w MRI acquired prior to implantation at the Chalfont Centre for Epilepsy, or ascertainable 2 year outcome. For the control cohort, healthy participants were also scanned at the Chalfont Centre for Epilepsy. Information on exclusion criteria can be found in Figure S1.

Individual response to VNS was defined as ≥50% reduction in seizure frequency at the clinical checkup closest to 2 years after VNS implantation, extracted from clinical letters.

### 2.2 T1w MRI acquisition and processing

#### 2.2.1 MRI acquisition

T1w MRI scans were acquired at the Chalfont Centre for Epilepsy using one of two 3 T GE scanners. Individuals had a fast spoiled gradient echo (FSPGR) scan at a resolution of .9375×.9375×1.1 mm, or magnetization‐prepared rapid acquisition gradient echo (MPRAGE) scan with a resolution of 1×1×1 mm. Exact acquisition parameters for the controls have been described previously.^17^ To help check that acquired scans were of sufficient quality to run through the HippUnfold pipeline, we first ran these through FreeSurfer, removing those that failed to complete or produced inaccurate outputs.

#### 2.2.2 HippUnfold

For each individual, we computed hippocampal volumetrics using HippUnfold's FreeSurfer parcellation.^18^ HippUnfold is a non–FreeSurfer‐associated package that provides an estimation of hippocampal subfield volumetrics using a U‐net machine learning model. We used the initial parcellation and combined regions to HippUnfold parcellation to the FreeSurfer package for analyzing hippocampal subfields^19^ containing the CA‐DG (cornu ammonis and dentate gyrus), SRLM (stratum radiatum, lacunosum, and moleculare), subiculum (including pre and post), and hippocampal tail.

#### 2.2.3 Univariate Z‐scoring

HippUnfold‐derived volumes were adjusted using ComBat to remove the effect of scan type (FSPGR or MPRAGE) while maintaining differences due to sex, age, and presence of epilepsy.^20^, ^21^ Volumes were then corrected for sex and age effects using a linear regression model trained on controls for each region. Volumes from all individuals were then z‐scored against controls on a per‐hemisphere, per‐region, and per‐subject basis.^22^, ^23^

#### 2.2.4 Multivariate Mahalanobis distance

We used the Mahalanobis distance^24^ to define abnormality relative to the control cohort within the hippocampus. For each individual i, their vector of z‐scores *Z*, were compared to the mean vector μ and covariance matrix C of healthy controls with the transpose operation T, resulting in the Mahalanobis distance M for the individual according to the following calculation:

$$M_{i}={\left[{\left(Z_{i}-\mu \right)}^{T}*C^{-1}*\left(Z_{i}-\mu \right)\right]}^{.5}$$

The Mahalanobis distance has been used as a measure of abnormality in epilepsy for MRI, electroencephalography (EEG), and magnetoencephalography (MEG) data.^15^, ^16^, ^25^ A higher Mahalanobis distance is indicative of a vector of values that differ from values seen in controls while accounting for the value covariance. Specifically, the Mahalanobis distance is sensitive to effects where one or more regions differ from the others in an unexpected way. Therefore, a high Mahalanobis distance is indicative of a regional abnormality, whereas a low Mahalanobis distance is indicative of a lack of regional abnormality. Such abnormality may reflect atrophy or hypertrophy, since the measure is agnostic to direction. An intuitive visual explanation of the Mahalanobis distance can be found in the study by Owen et al..^15^

#### 2.2.5 Defining the hippocampus of interest (HOI)

Here we define the hippocampus of interest (HOI) as being the hippocampus with the smaller mean z‐score across hippocampal regions on a per‐subject basis. This is a quantitative approach to finding the more atrophic hippocampus.

### 2.3 Statistical analysis

**FIGURE 1:**
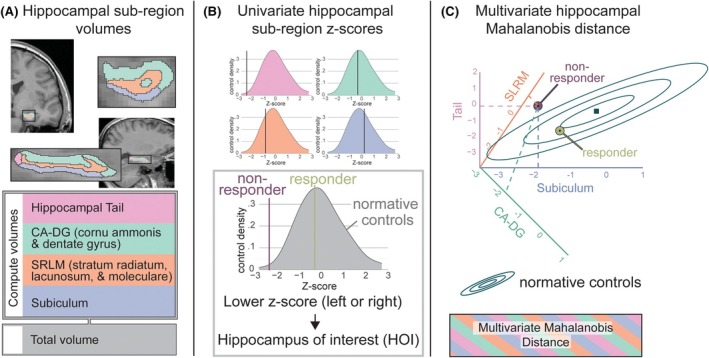
Methods summary: From T1‐weighted magnetic resonance imaging to structural hippocampal abnormality. (A) Structural volumetrics computed using HippUnfold in an individual. (B) For univariate volume comparisons, the individual volumes of the cornu ammonis and dentate gyrus (CA‐DG), stratum radiatum, lacunosum, and moleculare) (SRLM), subiculum, hippocampal tail, and whole hippocampus of interest (HOI) are z‐scored against controls. The hippocampus with the lower total volume z‐score is then selected as the HOI. (C) For multivariate comparison using the Mahalanobis distance, we take the four individual region z‐scores and combine them to calculate the Mahalanobis distance compared to controls.

All data analysis was performed using R version 4.3.2 (https://www.r‐project.org). To test the significance of demographic data, we used $\chi^{2}$ for categorical data and analysis of variance (ANOVA) for numeric data. To test the significance of quantitative T1w MRI data, we used a one‐tailed Wilcoxon rank‐sum test, as the normal distribution cannot be assumed. One‐tailed tests were used based on our prior hypothesis of greater abnormality in non‐responders.^4^ For effect sizes, the rank‐biserial correlation was used with associated qualitative labels.^26^ Figure 1 summarizes these methods.

## 3 RESULTS

### 3.1 Cohorts and demographics

From the 290 individuals with epilepsy and an implanted vagus nerve stimulator, 92 had T1w MRI acquired prior to implantation at the Chalfont Centre for Epilepsy and ascertainable 2 year outcome. Forty‐two individuals (46%) responded to VNS; 53 individuals (58%) with epilepsy were female. Age at time of MRI scan ranged from 17 to 71 years (median = 36 years, interquartile range [IQR] = 17 years). Duration of epilepsy varied from 3 to 67 years (median = 29 years, IQR = 18 years, three individuals had unknown duration of epilepsy) (Table 1).

**TABLE 1:** Demographic information for individuals with DRE and healthy controls.

Demographic	Non‐responders	Responders	Controls	Test statistic
Sex, female:male	33:17	20:22	62:38	$\chi^{2}$ = 4.65, *p* = .199
Age at scan, year	39 (17.8)	32.5 (17.8)	39 (21)	*F* = 2.45, *p* = .092
Scan type, FSPGR:MPRAGE	30:20	26:16	30:70	$\chi^{2}$ = 18.5, *p* < .001
Duration, year	28 (18.5)	23 (15.5)	NA	*F* = 1.28, *p* = .203
Scan gap, year	2.4(2.5)	3.3 (5)	NA	*F* = −1.86, *p* = .066
MRI lesion presence, yes:no	31:18	16:25	NA	$\chi^{2}$ = 4.33, *p* = .037
Hippocampal sclerosis, yes:no	13:37	3:39	NA	$\chi^{2}$ = 4.41, *p* = .036
Locational diagnosis, TLE:ETLE	25:14	13:11	NA	$\chi^{2}$ = .27, *p* = .605

Abbreviations: TLE, temporal lobe epilepsy; ETLE, extratemporal lobe epilepsy.

The control cohort included 100 healthy participants, of which 62 (62%) were female with age at MRI scan between 19 and 66 (median = 39, IQR = 21). A greater proportion of controls than individuals with epilepsy had MPRAGE T1w acquisitions (p>.001). Lesion presence on structural MRI was also significantly associated with negative outcome to VNS (p=.037, two individuals had no MRI diagnosis), which is contested in the literature.^4^, ^6^, ^7^, ^8^, ^9^, ^10^

Non‐responders were more likely to have a diagnosis of hippocampal sclerosis (HS;p=.036). Due to this difference between response groups and our focus on the hippocampus, we have displayed these results, excluding those with HS in Figure S3 to demonstrate consistency in our findings across both groups.

### 3.2 Multivariate HOI abnormality differs significantly between response groups

After determining the hippocampus with the smallest volume (the HOI), the Mahalanobis distance was calculated relative to controls in each individual using the four HOI regions. This multivariate distance measure is sensitive to different abnormalities in different individuals.^15^, ^16^ Information on rates at which HOI laterality corresponded with known lesion laterality are available in the supplementary materials.

**FIGURE 2:**
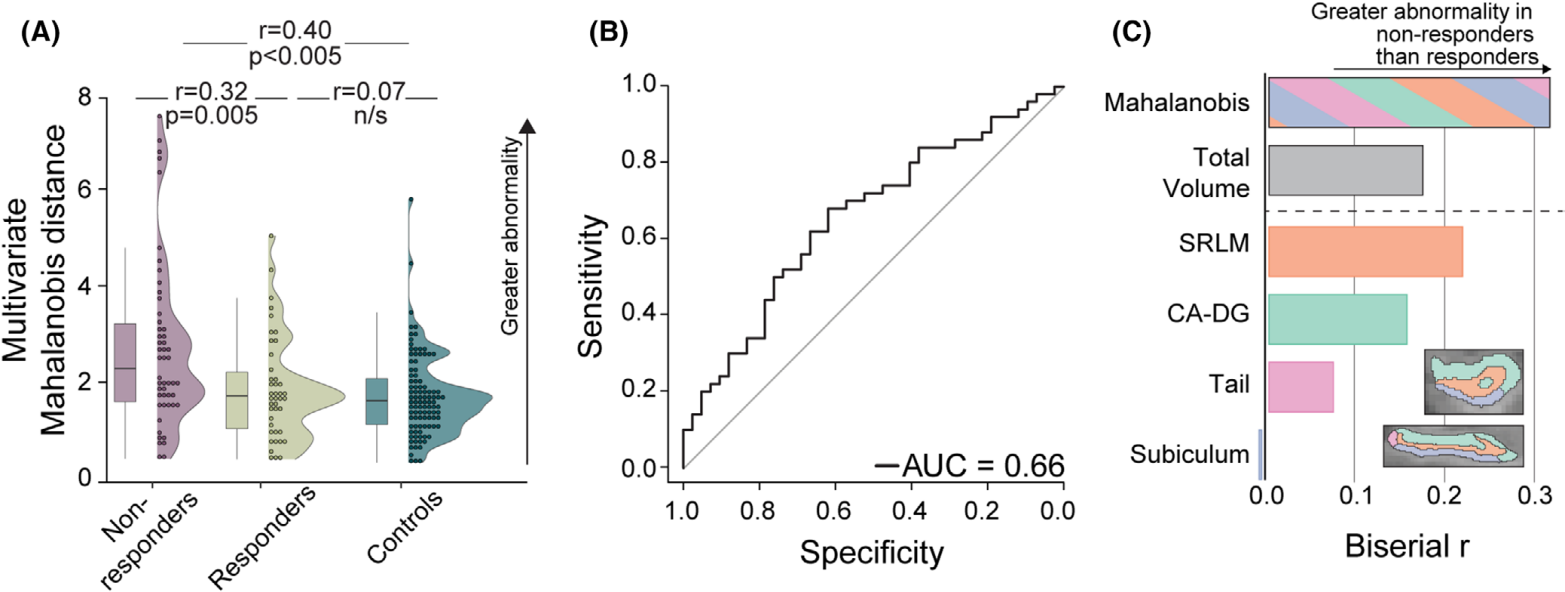
Hippocampal alterations and vagus nerve stimulation (VNS) response. (A) Non‐responder Mahalanobis distances were significantly greater than those of responders and controls using a Wilcoxon rank‐sum test (p=.005, r=.32). Responders and controls did not differ significantly (p=.273,r=.07). (B) A receiver‐operating characteristic (ROC) curve produces an area under the curve (AUC) between responders and non‐responders of .66. (C) Individual region abnormality does not vary significantly at the group level. Some regions showed differences between responders and non‐responders, but statistical significance is lost with multiple comparison corrections. Specifically, the hippocampus of interest (HOI) stratum radiatum, lacunosum, and moleculare (SRLM) showed a medium effect size but was insignificant after correction (p=.037, $p_{\mathrm{FDR}}=.147$, $p_{\mathrm{Bon}}=.147$). The harmonic p also indicated that the distribution of *p* values was not significant ($p_{\text{harmonic}}=.093$). Whole HOI volume did not differ significantly between response groups (p=.08, r=.17). Comparatively, the Mahalanobis distance showed a significant difference with the largest effect size.

Non‐responders had larger Mahalanobis distances to controls than responders p<.001,r=.40 for non‐responders to controls and p>.05,r=.07 for responders to controls respectively; Figure 2A. This effect was sufficiently large to result in a significant difference between non‐responders and responders (p=.005,r=.32). The receiver‐operating characteristic (ROC) curve had an area under the curve (AUC) of .66 (Figure 2B).

### 3.3 Univariate abnormality does not adequately delineate response

We next investigated if a simpler univariate measure of whole hippocampal volume explained response (Figure 2C).

The total HOI volume demonstrated a small, nonsignificant effect size and poorer AUC when comparing responders and non‐responders (r=.17,AUC=.59). This finding is presented as the gray bar in Figure 2C, with the earlier multivariate result plotted above as the striped bar for effect size comparison. Including both hippocampi did not improve group separation (Figure S2).

We then investigated if any of the hippocampal sub‐regions in isolation explained the response (Figure 2C). Larger bars demonstrate larger effects. No significant difference between responders and non‐responders was found. The largest effect was found in the SRLM (r=.22); however, this was not significant after correction for multiple comparisons ($p_{\mathrm{FDR}}=.147$). The CA‐DG showed a small effect size (r=.16), whereas the HOI tail and subiculum demonstrated minimal differences.

## 4 DISCUSSION

In this study we investigated if structural abnormality within the hippocampus was negatively correlated with response to VNS. The Mahalanobis distance, which combines information in different regions showed significantly higher abnormality for non‐responders compared to both responders and controls (p=.005, r=.32 and p<.001, r=.40, respectively). In contrast, more simple univariate approaches did not differ significantly between response groups. Overall, the multivariate Mahalobis distance, which is sensitive to abnormality in different regions in different individuals, demonstrated superiority over simpler univariate approaches.

The hippocampus is connected directly to the thalamus, amygdala, and brainstem nuclei, which are directly affected by afferent fibers from the vagus nerve, and are all key components of the vagus afferent network (VagAN).^12^, ^14^, ^27^ Hippocampal abnormality is a common finding in epilepsy and has been associated previously with variation in VNS response.^13^, ^14^ Specifically, Mithani et al.^14^ reported that white matter functional anisotropy more resembled controls in responders than non‐responders. Furthermore, Zhu et al.^13^ found that functional connectivity between the hippocampus and other VagAN regions varied between VNS responders and non‐responders but did not compare to controls. We extend this work by showing that structural hippocampal gray matter is altered in epilepsy non‐response groups compared to both controls and responders. In addition, clinically identified lesion presence was significantly negatively correlated with response to VNS, which is contested in the literature.^4^, ^6^, ^7^, ^8^, ^9^, ^10^

Univariate methods did not identify hippocampal abnormalities but would not be expected to do so if the mechanisms for non‐response were heterogeneous with regard to the part of the hippocampus involved. For example, abnormality in the CA‐DG might cause non‐response for one individual, whereas abnormality in the SRLM could be the case for another. Hippocampal abnormality is diverse across this cohort but uniform in relation to response, demonstrating that multivariate measures provide better clinical interpretation for variation in VNS response. Therefore, it is potentially useful that we do not just consider overall hippocampal volume differences but also localized volume differences.

The Mahalanobis distance combines regional information to provide a measure of overall abnormality. Using the Mahalanobis distance, we found that non‐responders differed significantly from responders and controls. This outperforms univariate measures as it considers both total abnormality and variation in regional abnormality for all individuals. Overall, the Mahalanobis distance allows for comparisons between varied abnormalities allowing for better isolation of hippocampal abnormality.

Although we find a significant effect of hippocampal abnormalities on VNS response, this is driven by a select few individuals who display large amounts of hippocampal abnormality beyond that seen in the control cohort. This leads to a limited AUC in terms of outcome prediction (AUC = .66). However, this is expected, as structural hippocampal abnormality is one of many reasons we expect to cause VNS non‐response. This non‐response association with abnormality beyond control values supports the idea of a point of maximal abnormality where VNS loses its effectiveness, as discussed previously.^4^ Abnormalities beyond the hippocampus, whether those be structural or otherwise, will also contribute to this maximal point of abnormality and are valuable targets for future research.

Furthermore, we find that outcomes do not vary between individuals diagnosed with temporal and extratemporal lobe epilepsy as shown previously^10^, ^28^ (Data S1). This demonstrates that hippocampal volumetrics are valuable in both groups and should be considered in future studies. Comparatively, diagnosis of hippocampal sclerosis is associated with non‐response in our cohort, which should be considered in further studies.

T1w MRI is acquired for almost all individuals with DRE. As such, any VNS response marker from T1w imaging could be widely used, with little to no marginal cost. Furthermore, the high spatial resolution and interpretability provides the opportunity to improve our mechanistic understanding should a marker be identified. Additional predictors found with other modalities could also provide insight, which should be the goal of future research. For example, EEG, MEG, DWI, and functional MRI (fMRI) have been suggested as possible markers of response, and could be combined with T1w MRI markers to improve response prediction,^14^, ^27^, ^29^, ^30^, ^31^, ^32^ although systematic availability of these modalities is less common in clinical cohorts. In addition, we believe that signal intensities in fluid‐attenuated inversion recovery (FLAIR) imaging, which is commonly acquired clinically, would also provide valuable information about both the hippocampus and other brain regions. A multi‐modal approach integrating T1w MRI and other modalities may be most insightful.

### 4.1 Limitations and future work

Individuals who receive VNS are often those where a singular epileptogenic zone could not be found, or who are deemed unsuitable for resective surgery (such as for having an epileptogenic zone within the eloquent cortex). Therefore, the population of individuals with epilepsy treated with VNS is not representative of epilepsy as a whole. Regardless, our study is representative of individuals receiving VNS in the real world, in our clinical center. The single‐center limitation could be addressed with a cross‐site comparison using normalization techniques such as those done previously with other brain metrics.^33^

Due to the retrospective nature of this study, VNS response was ascertained from clinical notes primarily using seizure diaries, which may not be accurate.^34^, ^35^, ^36^ In addition, this binary outcome does not provide a holistic view of response, as individuals with significant benefits to seizure duration, severity, mood, and quality of life measures could be characterized as “non‐responders” using the ≥50% seizure reduction criterion. Nonetheless, seizure reduction in VNS has been associated previously with better quality of life.^37^

## 5 CONCLUSION

VNS non‐responders had significantly more abnormal multivariate hippocampal morphometrics than responders. This suggests that (1) greater structural hippocampal abnormality is associated with poorer VNS efficacy and (2) the origin of this abnormality is heterogeneous between individuals. These results reflect that abnormalities gathered from T1w MRI is useful in delineation of response groups to VNS, and, specifically, that hippocampal damage could be considered when offering VNS to individuals with DRE.

## AUTHOR CONTRIBUTIONS

**Harry J. Clifford, Peter N. Taylor, and Sonja Fenske:** Conceptualization, formal analysis, investigation, and methodology. **Sabahat Iqbal and Cameron A. Elliot:** Data acquisition from hospital records. **All authors:** Interpretation of data for the work, drafting the work or revising it critically for important intellectual content, final approval of the version to be published, and agreement to be accountable for all aspects of the work in ensuring that questions related to the accuracy or integrity of any part of the work are appropriately investigated and resolved.

## FUNDING INFORMATION

T.d.S.C. is supported by an National Institute for Health and Care Research (NIHR) Newcastle Biomedical Research Centre Fellowship; P.N.T. and Y.W. are supported by UKRI Future Leaders Fellowships (MR/T04294X/1, MR/V026569/1).

## CONFLICT OF INTEREST STATEMENT

None of the authors has any conflict of interest to disclose.

## ETHICS STATEMENT

We confirm that we have read the Journal's position on issues involved in ethical publication and affirm that this report is consistent with those guidelines.

## Supporting information


Data S1.

## Data Availability

Anonymized data and code to reproduce the findings of this study are at the following location https://github.com/cnnp‐lab/mahal‐vns.
