# Supplementary material for: Hippocampal abnormality and response to vagus nerve stimulation in epilepsy
Source: Epilepsia. 2025 Oct 3;66(12):e268–75. doi: 10.1111/epi.18658 (PMC12779329; doi:10.1111/epi.18658)
Supplement: Supplementary file 1 — Data S1. [file EPI-66-e268-s001.docx]

Hippocampal abnormality and response to Vagus Nerve Stimulation in epilepsy

Supplementary Materials

Contents

[Supplementary Figure 1: Inclusion Criteria 2](#_Toc207045278)

[Supplementary Figure 2: Whole HOI and Combined hippocampi analyses 4](#_Toc207045279)

[Supplementary Figure 3: Analysis excluding individuals with hippocampal sclerosis 5](#_Toc207045280)

[Supplementary Figure 4: Analysis of MRI positive without HS 6](#_Toc207045281)

[Supplementary Data 1: Effect of locational diagnoses (ETLE/TLE) 6](#_Toc207045282)

[Supplementary Data 2: HOI lateralities correspondence with known lesion laterality 7](#_Toc207045283)

[Supplementary Data 3: Summary of lesion type 7](#_Toc207045284)

Supplementary Figure 1: Inclusion Criteria


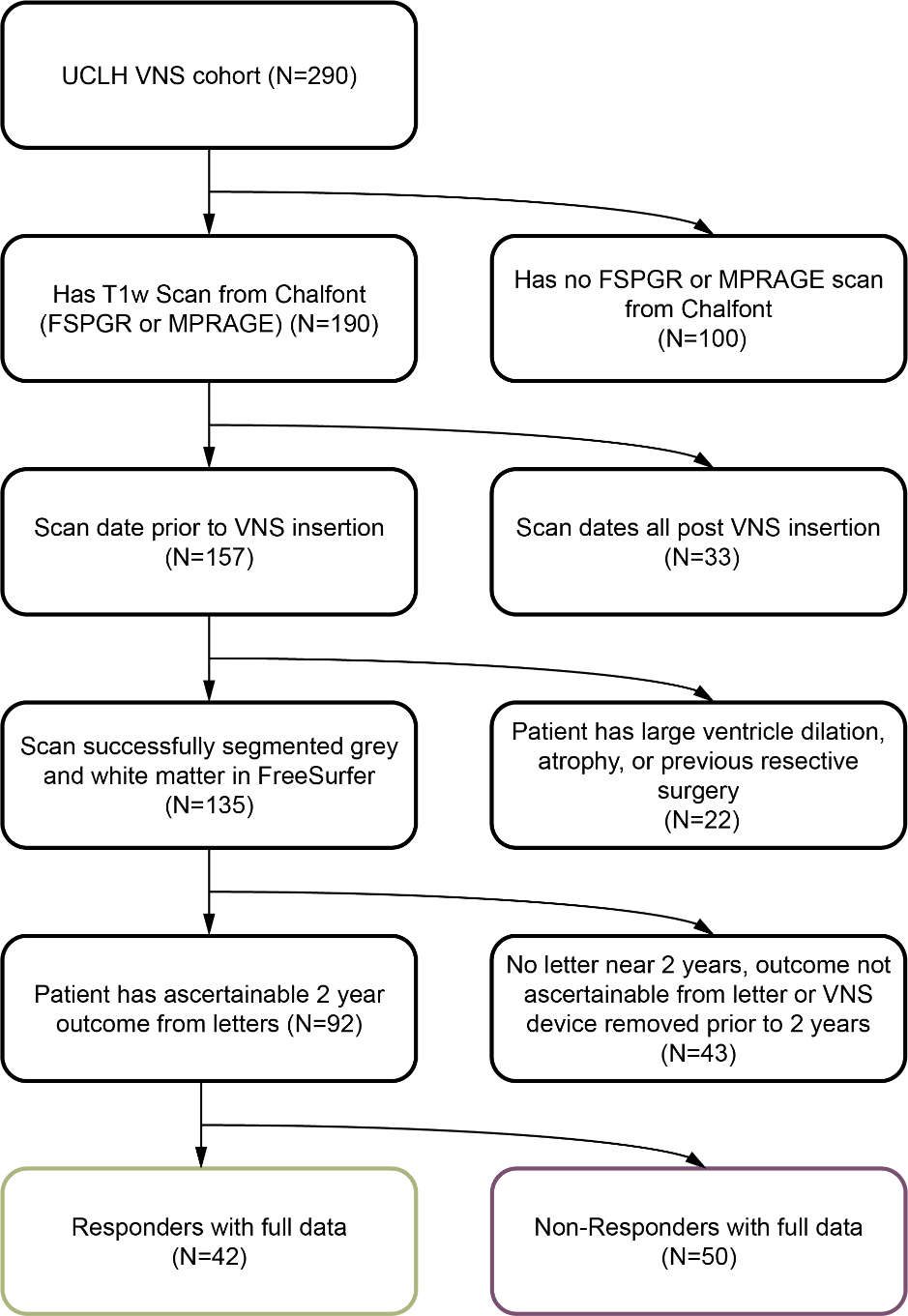


**Supplementary Figure 1: Reasons for exclusion from analysis.** We collected demographic details of 290 individuals with VNS implantations from the Chalfont Centre for Epilepsy. 198 individuals were excluded due to either; a lack of appropriate T1w MRI scan prior to implantation, large scale atrophy or prior resection which make quantitative analysis tools questionable, and lack of ascertainable outcome 2 years after VNS implantation from clinical notes. This left 92 individuals with Epilepsy for analysis (42 Responders and 50 Non-Responders)

# Supplementary Figure 2: Whole HOI and Combined hippocampi analyses


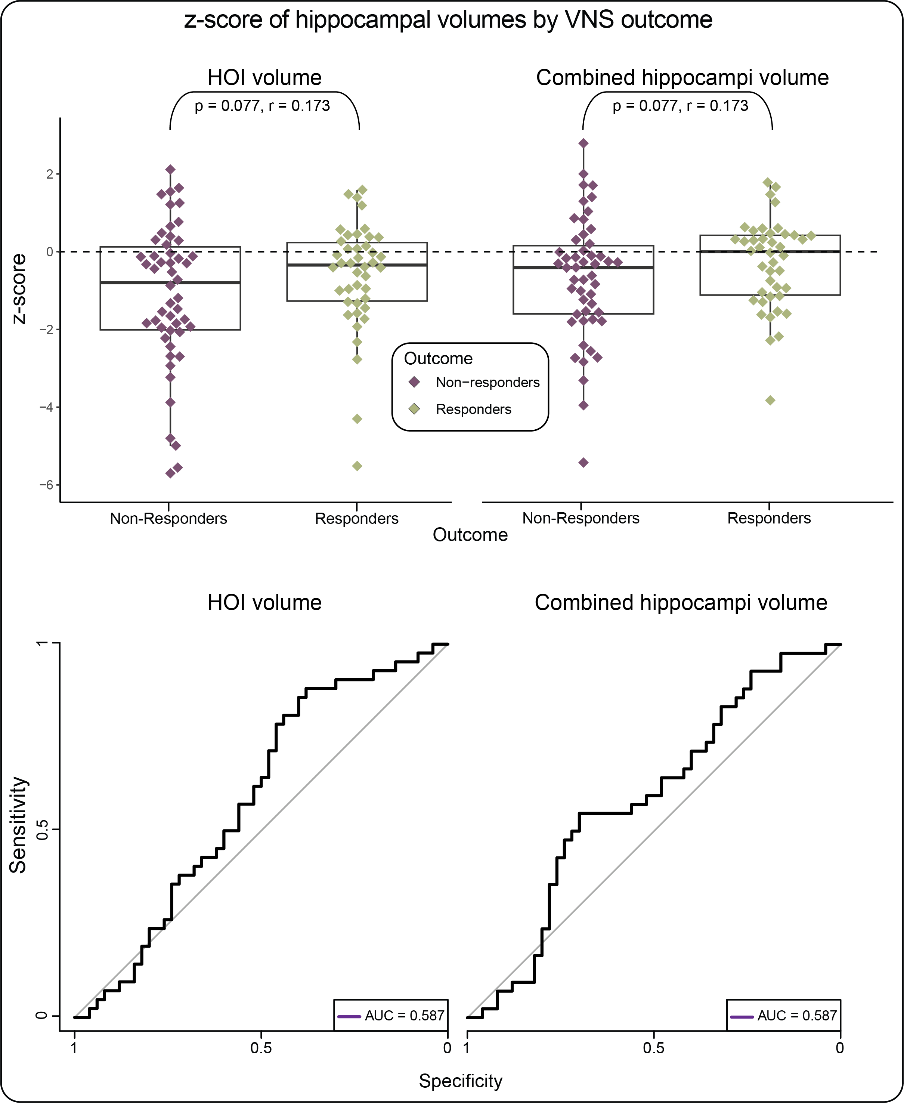


**Supplementary Figure 2: Differences in outcome to VNS cannot be ascertained using whole hippocampal morphometrics.** A small relationship between the volume in the HOI and combined hippocampal volume to VNS response was seen. However, this is insignificant at p = 0.05 with a small effect size. Additionally, a ROC curve produced using these measures performs poorly at predicting outcomes (AUC = 0.587 for both measures).

# Supplementary Figure 3: Analysis excluding individuals with hippocampal sclerosis


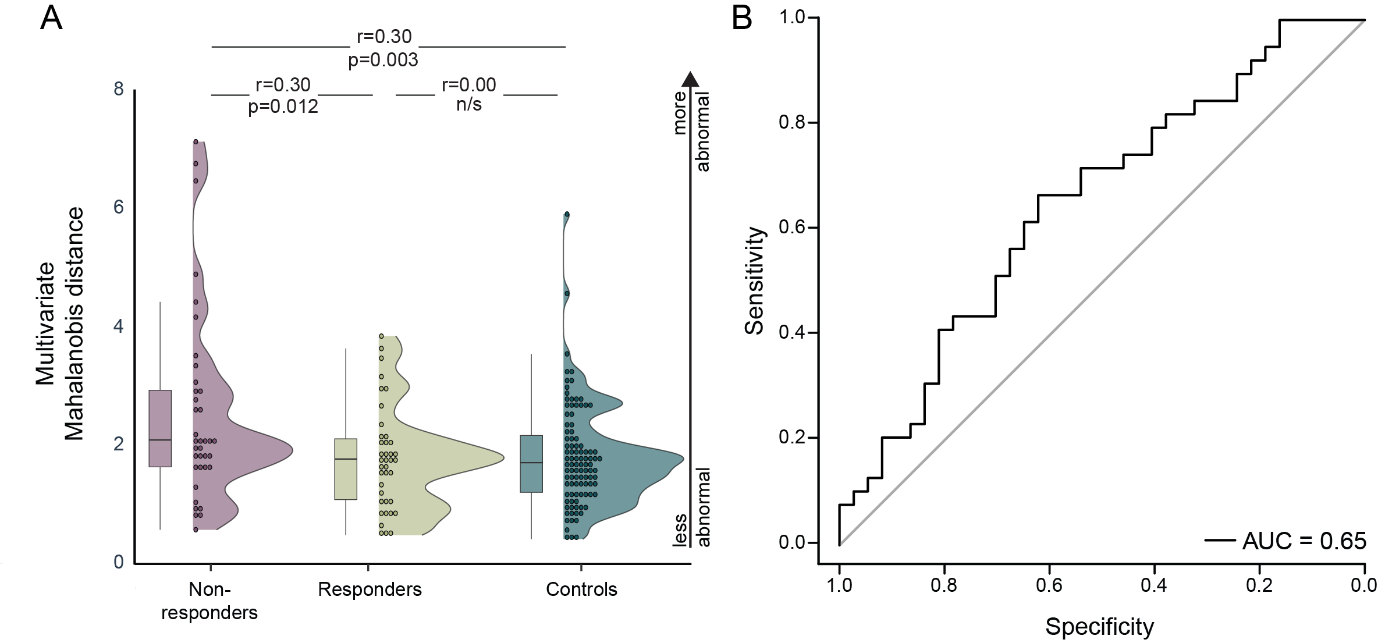


**Supplementary Figure 3: Results remain consistent when only considering individuals without clinically marked HS.** When considering individuals without MRI diagnosed HS, effect sizes are reduced between Non-responders and Controls (r=0.3, p=0.003). However, effect sizes remain consistent between Non-responders and Responders (r=0.3, p=0.012) along with the AUC (0.65). This demonstrates that hippocampal abnormality beyond visually identified HS is present in VNS non-responders.

# Supplementary Figure 4: Analysis of MRI positive without HS


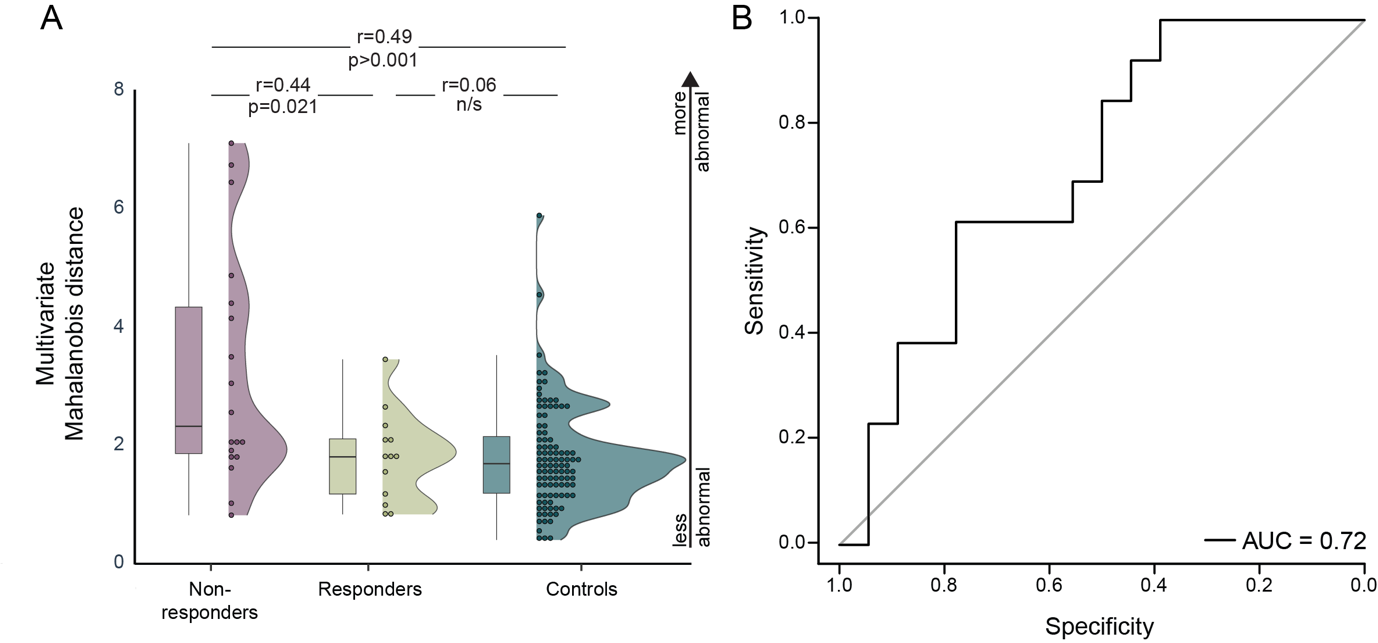


**Supplementary Figure 4: Results remain consistent when only considering individuals with an identified lesion which is not HS.** When considering individuals with non-HS lesions, effect sizes are increased between Non-responders and Controls (r=0.49, p<0.001). Along with effect sizes between Non-responders and Responders (r=0.44, p=0.021) along with the AUC (0.72). This demonstrates that hippocampal abnormality beyond visually identified HS for those with other MRI identified lesions is present in VNS non-responders.

# Supplementary Data 1: Effect of locational diagnoses (ETLE/TLE)

|  | | Localisation | | |
| --- | --- | --- | --- | --- |
|  |  | TLE | ETLE | Unknown |
| Outcome | Non-responder | 25 | 14 | 11 |
|  | Responder | 13 | 11 | 18 |

Chi^2^ p = 0.075

Chi^2^ p excluding unknown = 0.605

Differences in seizure onset localisation are insignificant between response groups. Specifically, responders are more likely to have an unknown seizure onset which is likely due to their increased likelihood of an MRI- diagnosis. When removing these individuals to remove this confound, we find an even smaller effect which fails to approach significance.

Additionally, we performed the main analysis using the Mahalanobis distance on these subgroups. Individuals with TLE show significant differences (p = 0.04) between responders and non-responders with a large effect size (r = 0.35). Comparatively both ETLE and unknown groups failed to hold significance with only a moderate effect size (p = 0.20, r = 0.21 and p = 0.20, r = 0.20 for ETLE and unknown respectively). This is likely due to the increased number of individuals who are MRI positive and/or have HS within the TLE subgroup driving this effect.

# Supplementary Data 2: HOI lateralities correspondence with known lesion laterality

|  | | Lesion laterality | | | |
| --- | --- | --- | --- | --- | --- |
|  |  | Left | Right | Bilateral | Unknown/NA |
| HOI laterality | Left | 11 | 5 | 8 | 33 |
|  | Right | 0 | 12 | 2 | 21 |

HOI laterality corresponds with the location of known seizure foci. Of those with a known unilateral seizure focus 23/28 had the same side indicated as the HOI. In general, individuals were identified as having a more abnormal left hippocampus even when their known seizure foci were unknown or bilateral. This demonstrates that our method of defining the HOI supports underlying expectation based on known seizure foci.

# Supplementary Data 3: Summary of lesion type

Individuals had a wide range of aetiologies and pathologies, including hippocampal sclerosis (n=16), focal cortical dysplasia (n=7), glioma (n=4), cyst (n=3), ischemia (n=2), heterotopia (n=2), traumatic brain injury (n=2), encephalitis (n=2), and other pathology (n=9). 43 individuals had no lesion clically visible on MRI, and 2 had no specific lesion listed.
